# Nutritional Rickets and Osteomalacia in the Twenty-first Century: Revised Concepts, Public Health, and Prevention Strategies

**DOI:** 10.1007/s11914-017-0383-y

**Published:** 2017-06-13

**Authors:** Suma Uday, Wolfgang Högler

**Affiliations:** 1 Department of Endocrinology and Diabetes, Birmingham Children’s Hospital, Steelhouse Lane, Birmingham, B4 6NH UK; 2 Institute of Metabolism and Systems Research, University of Birmingham, Birmingham, UK

**Keywords:** Nutritional rickets, Osteomalacia, Vitamin D, Supplementation policy, Food fortification, Dietary calcium

## Abstract

**Purpose of Review:**

Nutritional rickets and osteomalacia are common in dark-skinned and migrant populations. Their global incidence is rising due to changing population demographics, failing prevention policies and missing implementation strategies. The calcium deprivation spectrum has hypocalcaemic (seizures, tetany and dilated cardiomyopathy) and late hypophosphataemic (rickets, osteomalacia and muscle weakness) complications. This article reviews sustainable prevention strategies and identifies areas for future research.

**Recent Findings:**

The global rickets consensus recognises the equal contribution of vitamin D and dietary calcium in the causation of calcium deprivation and provides a three stage categorisation for sufficiency, insufficiency and deficiency. For rickets prevention, 400 IU daily is recommended for all infants from birth and 600 IU in pregnancy, alongside monitoring in antenatal and child health surveillance programmes.

**Summary:**

High-risk populations require lifelong supplementation and food fortification with vitamin D or calcium. Future research should identify the true prevalence of rickets and osteomalacia, their role in bone fragility and infant mortality, and best screening and public health prevention tools.

## Introduction

The incidence of nutritional rickets (NR) is rising globally [1], and hospitalisation is increasing even in high income countries [2••, 3]. The underlying calcium deprivation [4•] does not just manifest as reduced bone mineralisation (rickets and osteomalacia) but also as hypocalcaemic seizures, tetany and dilated cardiomyopathy including cardiac failure and death [5, 6, 7•]. Undiagnosed NR has also been implicated in childhood mortality [8•, 9]. The prevalence of osteomalacia histologically at post-mortem in adult Europeans is as high as 25% [10•]. Therefore, clinically evident NR is only the tip of the iceberg, and the true burden of subclinical rickets and osteomalacia remains unidentified. Calcium deprivation is caused by two factors, low dietary calcium and vitamin D intake. Vitamin D deficiency is pandemic in Europe [11•], in winter affecting nearly 18% of the population, but with 3–71 times higher risk in dark-skinned ethnic minority groups [11•]. The increasing prevalence of vitamin D deficiency mirrors the trends in NR, dark-skinned individuals being at a higher risk [12••].

## Rickets and Osteomalacia—Definition, Risk Groups and Terminology

### Definition

Rickets constitutes the defective mineralisation of growth plates [13••, 14, 15]. The underlying mechanism of all forms of rickets is low serum phosphate resulting in reduced apoptosis of hypertrophic chondrocytes in the growth plate and reduced mineralisation of primary spongiosa in the metaphysis (new bone) [16]. In NR, hypophosphataemia is created by secondary hyperparathyroidism (see below). Osteomalacia constitutes defective mineralisation of existing (old) bone during the remodelling process and therefore always goes along with rickets in growing children (open growth plates) and occurs ubiquitously in bones of adults or adolescents (closed growth plates). Therefore, osteomalacia is not just a disease of adults, but the main reason for long bone bowing deformities and fractures in children with rickets, as poor mineralisation reduces bone stiffness. Low calcium intake and/or low vitamin D (from lack of sunshine exposure) are the leading causes of body calcium deprivation worldwide and their combined deficiency accelerates bone demineralisation [13••, 14, 17].

### Risk Groups

Public health research has identified traditional diets low in calcium, dark skin and cultural full body clothing, as the predominant causes of rickets and osteomalacia in sunny parts of the world such as the Indian subcontinent [18•, 19], the Middle East [20] and Africa [21]. In high Northern or Southern latitudes (more than approximately 34°), it is the seasonal lack of the ultraviolet-B (UV-B) spectrum of sunlight that causes seasonal vitamin D deficiency (also called ‘vitamin D winter’) [11•]. In high latitude countries, the dark-skinned immigrant and resident population is at greatest risk [12••].

### Terminology De-confused

Vitamin D (calciferol) is made in the human skin (D3) following exposure to UV-B light, or ingested with food (D2 and D3), then converted in the liver to 25-hydroxyvitamin D (25OHD, calcidiol) and finally in the kidney and also the gut [22] to the active hormone 1,25(OH)_2_D (calcitriol or ‘active vitamin D’). Sadly, this terminology has confused generations of healthcare professionals. Even today, conference presenters refer to calciferol, calcidiol and calcitriol as ‘vitamin D’. To add to the confusion, all three steroids exist either in their D2 (ergo-) or D3 (chole-) form, not to mention other, poorly explored secosteroid versions of this vitamin. Vitamin D and 25OHD are biologically inert, whereas calcitriol is the active, potent hormone with its main function to increase intestinal absorption of calcium and phosphate. Experts in adrenal and sex-steroids are well used to dealing with steroid terminology, various hydroxylation steps, and the concept of inert and active metabolites. In analogy to other steroid pathways, cortisol is not ‘active cholesterol’, or the mineralocorticoid receptor is not a ‘cholesterol receptor’. Vitamin D does not bind to the ‘vitamin D receptor’, only calcitriol does and hence the appropriate term for this receptor is ‘calcitriol receptor’. This is not a semantic discussion. Even in the twenty-first century, there are reports where calcitriol is used incorrectly to replace severe vitamin D deficiency, which not only leaves the individual vitamin D deficient but worsens the situation by suppressing 25-hydroxylase activity and risking hypercalcaemia. The correct replacement for vitamin D deficiency is vitamin D (calciferol). What is measured in the blood stream as the best marker of vitamin D status is not vitamin D, but 25OHD. It is time to reconsider the terminology we use and move forward into the twenty-first century with precise and self-explanatory terms.

## Calcium Deprivation: Concept and Clinical Spectrum

### The Concept of Calcium Deprivation

**Fig. 1:**
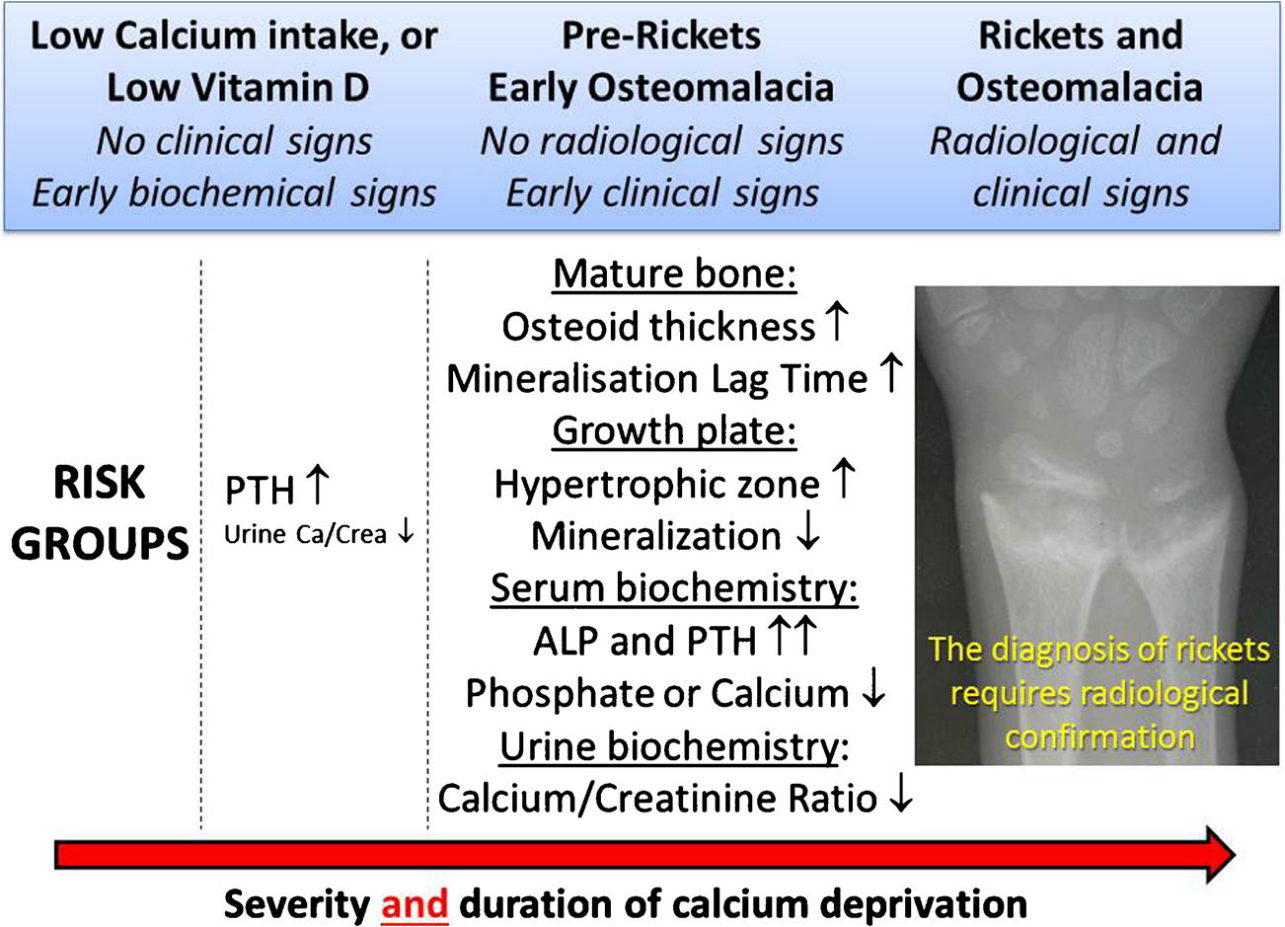
Stages of calcium deprivation leading to nutritional rickets and osteomalacia

Calcium and phosphate are the main mineral ions responsible for bone stiffness. Calcitriol is the main supplier of these bone minerals, which are ingested daily, by increasing their intestinal absorption. Calcitriol therefore cannot do its job in individuals who have no oral intake. Combined or in isolation, low dietary calcium supply and low vitamin D status cause calcium deprivation, which is quickly sensed by the calcium sensing receptor in the parathyroid’s chief cells, leading to increased release of parathyroid hormone (PTH) [23]. This secondary hyperparathyroidism stimulates osteoclastic bone resorption with the aim to release stored bone minerals. This compensation mechanism works well to maintain normal serum calcium levels, but sustained hyperparathyroidism has two main complications: (1) structural damage to bone and (2) decreased renal reabsorption of phosphate. Eventually, this compensation will fail as the calcium stored in bone will ultimately be depleted, leading to *hypocalcaemic complications* (seizures, tetany and cardiomyopathy) and late *hypophosphataemic complications* (nutritional rickets, osteomalacia and muscle weakness) unless more calcium is made available to restore eucalcemia and normalise PTH levels. Hypophosphataemia increases the formation of poorly mineralised osteoid (osteomalacia) alongside a rise in serum alkaline phosphatase (ALP) and causes the growth plate abnormality known as rickets. The concept of calcium deprivation and its evolution into rickets and osteomalacia is depicted in Fig. 1. Because secondary hyperparathyroidism maintains normal serum calcium levels until compensation fails, serum calcium is a poor marker of calcium status, whereas 25OHD is currently regarded as the best marker of vitamin D status. Therefore, the earliest biochemical signs of clinically relevant calcium deprivation are increased serum PTH and decreased urinary calcium levels; both are sensitive markers of total body calcium deprivation and will provide an indicator of both the severity and duration of the vitamin D deficient or the dietary calcium deficient state.

### The Clinical Spectrum of Calcium Deprivation from Conception to Old Age

The role of vitamin D as a mineral supplier in normal bone and dental development is well established [24]. Maternal calcium deprivation rarely affects the foetus who is protected by drawing minerals from the mother through increased maternal intestinal mineral absorption and bone resorption [25]. However, low maternal vitamin D reserve is always passed on to the foetus. Hence, vitamin D supplementation needs to start at birth to protect the neonate from developing complications from calcium deprivation, including rickets, during the rapid infantile growth [4•]. Depending on the duration and severity of maternal vitamin D deficiency, the unsupplemented infant is at high risk of presenting in the first few days or months of life with hypocalcaemic complications. These include seizures, tetany [7•, 26, 27] and dilated cardiomyopathy which may result in cardiac failure and death [5, 6, 28]. Hypophosphataemic complications include muscle weakness leading to hypotonia and delayed development, craniotabes, large fontanelles and bony deformities which usually present in the first 18 months of life [29, 30]. Only severe, long-standing calcium deprivation during pregnancy will manifest in the newborn skeleton at birth, referred to as congenital rickets [31]. If undiagnosed, there is a concern that hypocalcaemia, cardiomyopathy and rickets will contribute to or cause infant mortality. A retrospective analysis post-mortem identified hypocalcaemia contributing to death in 3 of 52 children; two had cardiomyopathy and one had hypocalcaemic seizures. All three patients were of Black ethnic origin and also had histological and radiological evidence of rickets [8•]. Another UK post-mortem study highlighted that a significant proportion (76%) of children with unexplained sudden death (*n* = 25) had suboptimal vitamin D levels, of whom 69% had histological signs of rickets [9]. In infants and young children, rickets remains a radiological diagnosis that needs to be confirmed by taking a knee X-ray.

Older children can present with motor delay, proximal myopathy [32] and dental complications [33]. As NR frequently manifests in phases of rapid growth, a second peak in incidence of hypocalcaemic seizures and NR is seen in adolescence [3, 7•]. Diagnosis is based on clinical findings (long bone deformities, enlargement of wrists and costochondral junctions), typical biochemical abnormalities (increased ALP and PTH) and radiological changes of rickets (cupping, splaying and fraying of metaphyses, widened growth plates and low bone mass). In older children and specifically adolescents with less rapid growth, the radiological changes of the growth plate typical for rickets may not be visible, and equally, osteomalacia cannot be diagnosed on X-rays.

Osteomalacia in girls can cause pelvic deformities and lead to obstructed labour later in life [34]. Elderly and institutionalised individuals develop muscle weakness which manifests as increased falls and fractures, the incidence of which is reduced with calcium and vitamin D supplements [35].

Manifestations of all these complications of calcium deprivation throughout life demonstrate that dietary calcium and vitamin D deficiency are widespread in society, particularly among high-risk groups.

## Sufficient Dietary Calcium and Vitamin D, and Treatment of Rickets

### How Much Vitamin D and Dietary Calcium is Enough?

The ‘optimal’ serum 25OHD concentration remains controversial, with definitions ranging from >50 to >100 nmol/L (20 to 40 μg/L) [36]. Multiple paediatric and endocrine societies have independently made recommendations for deficient and sufficient serum 25OHD levels [37–41]. Disagreement among societies [42] creates confusion among healthcare professionals, researchers and the general public. A good part of the confusion stems from the ignorance of vitamin D enthusiasts towards the role of dietary calcium intake and PTH in the pathophysiology of rickets and osteomalacia. To provide clarification on this issue, representatives from 11 international scientific organisations came together in 2014 to create evidence-based Global Consensus Recommendations on Prevention and Management of Nutritional Rickets [13••, 14]. These papers treat calcium intake and vitamin D status equally and define each by a three-stage category as sufficient, insufficient and deficient. The term ‘sufficiency’ in the consensus was based on minimum calcium intakes and 25OHD levels required to prevent NR **(**Table 1 **)**.

**Table 1:** Global consensus definitions of vitamin D status and dietary calcium intake [13••, 14]

	Serum 25OHD levels	Daily calcium intake
Deficient	>50 nmol/L	>500 mg
Insufficient	30–50 nmol/L	300–500 mg
Sufficient	<30 nmol/L	<300 mg
Evidence grade	*1* ***⊕ ⊕ ⊕*** ^a^	*1* ***⊕ ⊕*** ^b^

^a^Strong recommendation with high quality evidence

^b^Strong recommendation with moderate quality evidence

### Management of NR

The Global Consensus group recommended doses of vitamin D and calcium for treatment of NR [13••, 14]. Evidence indicates that the oral route is preferred to parenteral (Stoss therapy) due to more rapid restoration of 25OHD levels. Both vitamin D2 and D3 are equally effective for daily treatment, whilst D3 with a longer half-life is preferred for single dose treatment. All children with NR should be treated with vitamin D for a minimum of 3 months with a daily dose of at least 2000 IU (50 μg) if aged <12 months, 3000–6000 IU (50–150 μg) if aged 12 months–12 years, and 6000 IU (150 μg) if aged >12 years [13••, 14]. Single high dose (Stoss therapy) can be used in resource-limited settings in infants aged >3 months: 50,000 IU (1250 μg) for 3 months–12 months of age, 150,000 IU (3750 μg) for children aged 12 months–12 years, and 300,000 IU (7500 μg) if aged >12 years [13••, 14]. All individuals should also receive concomitant calcium (minimum 500 mg/day) as supplements or via diet. All treatment should be followed by lifelong vitamin D supplements, since the underlying risk (ethnicity, culture and sunlight exposure) is unlikely to change.

## Prevention and Public Health

### Identification of Risk Groups is Straightforward

In developed countries, the prevalence of NR is several hundred-fold higher in dark-skinned immigrants compared to native populations [12••]. This means conditions that are rare in the host country are common in ethnic minorities. The underlying endemic vitamin D deficiency in these ethnic risk groups is exemplified by a population-based study in 2225 school children and 830 adults in Saudi Arabia, demonstrating that 92% of girls and 79% of boys, and 75% and 74% of adult women and men, respectively, had 25OHD levels below 50 nmol/L (20 μg/L) [43•]. In developed countries, there has been a steady increase in the proportion of dark-skinned populations (both immigrant and resident) which mirror the trends in NR [12••]. Healthcare authorities should identify the specific risk groups to target intervention which is a straightforward task as skin colour, ethnicity and culture are visible factors; similarly, information on dairy intake as the main source of dietary calcium can be easily obtained [44]. Healthcare providers should recognise the need to supplement the high-risk individuals upon arrival into the country [12••], and supplementation should be incorporated into immigrant/refugee health assessment policies. The mounting evidence on endemic calcium deprivation and NR in risk groups precludes the need for routine 25OHD measurement in asymptomatic individuals; the consensus group therefore recommends lifelong supplementation in high-risk groups [13••, 14].

In developing countries populated by high-risk ethnic groups, the failure, or non-existence, of vitamin D supplementation policies are main contributors to the rise in NR despite abundant sunshine [18•, 45–47]. A recent study from Egypt reported a high incidence (13.1%) of rachitic genu varum in children aged 2–4 years [48]. The study reported the following risk factors to be associated with the development of NR: low socioeconomic status, insufficient family income, poor housing conditions, lack of sunlight exposure due to cultural practices, sole breast feeding and inadequate supplementation of vitamin D in children and pregnant women [48].

### Effective Prevention

NR and osteomalacia are fully preventable. Universal supplementation of infants [49], supplementing pregnant women [50], promoting vitamin uptake [51] and also food fortification with vitamin D and calcium to prevent fractures in elderly [52] have been proven cost-effective. However, there is limited data on vitamin D fortification and how fortification programmes compare to population supplementation programmes [53].

The global consensus recommends the following vitamin D supplements for *prevention* of NR and osteomalacia [13••, 14]:

- 400 IU (10 μg) daily for all infants regardless of mode of feeding, from birth to a minimum of 12 months of age.
- 600 IU (15 μg) daily during pregnancy (alongside iron and folic acid).
- 600 IU daily lifelong in risk groups, including individuals with dark skin, full body clothing, limited sun exposure either due to geographic location, limited outdoor activity or restricted mobility, low socioeconomic background and poor diet. Individuals at risk should also meet the daily minimum requirement for sufficient calcium intake (Table 1).

Effective prevention programmes depend on feasible, monitored and mandatory implementation strategies.

#### Monitored Supplementation of Pregnant Women

Low socioeconomic status, covered clothing and lack of supplementation contribute to high prevalence of vitamin D deficiency in pregnancy [54]. Congenital rickets and postnatal hypocalcaemic seizures are reported in risk groups in both developed and developing countries [27, 55, 56]. Poor maternal vitamin D status affects the foetus and the newborn. In a large Italian study, 76% of newborns of dark-skinned migrant women had 25OHD levels below 25 nmol/L (10 μg/L), compared to 38% of newborns of native Italian women [57•]. Although the World Health Organisation is yet to provide stronger recommendations, it confirms that vitamin D supplementation during pregnancy is necessary to prevent NR in the newborn [58]. The global consensus group recommends that monitored vitamin D supplementation should be included in antenatal care programmes [13••, 14].

#### Universal Supplementation of Infants Regardless of Mode of Feeding

The amount of vitamin D available in breast milk but also infant formula milk is insufficient to prevent NR; even formula-fed infants can present with symptomatic deficiency in the first few months of life [7•, 59, 60] if born to deficient mothers. In a recent survey of vitamin D supplementation policies across Europe, we found that universal supplementation, currently practised by 79% (23/29) of countries was significantly (*p* = 0.007) associated with good adherence to supplements [61]. The recommended dose of 400 IU/day (10 μg) is safe also in formula-fed infants. Toxicity is usually related to errors in manufacturing, formulation or prescription [62, 63]. A Dutch study pointed out the theoretical risk of exceeding the upper limit of vitamin D consumption in infants aged 7–11 months from a combination of infant formula, recommended supplements (10 μg/day) and fortified food [64], based on the upper limit of 1000 IU/day (25 μg), recommended by the European Food Safety Authority. However, none of the infants in the study exceeded the upper limit recommended by the Institute of Medicine which is 1500 IU/day (37.5 μg) [40].

#### Supplementation Beyond the First Year of Life

The duration of childhood supplementation varies widely across Europe [61]. Assessment of daily intakes at 18 months and 3.5 years of age in 755 children in the UK showed low daily intakes of vitamin D and calcium [65]. The incidence of NR rises beyond the recommended age of infant supplementation [3, 66, 67], especially in dark-skinned (immigrant or resident) individuals. Hence, national policies should ensure that the daily requirement of vitamin D beyond the first year of life is met through supplementation or fortification [68•]. In the absence of food fortification, policy makers should identify the high-risk ethnic groups and recommend lifelong supplementation (Table 2).

**Table 2:** Summary of revised concepts

Topic	Revised concepts
Calcitriol is not vitamin D	Like cholesterol is biochemically modified by the human body to form active steroid hormones, vitamin D is modified to form the hormone calcitriol. Calcitriol acts on the calcitriol receptor (VDR), whilst vitamin D and 25OHD are biologically inert.
Calcium deprivation and its complications	Calcium deprivation occurs secondary to low dietary calcium and/or low vitamin D. Calcium deprivation has hypocalcaemic (seizures, tetany and cardiomyopathy) and late hypophosphataemic (rickets, osteomalacia and muscle weakness) complications.
What to measure and how to make a diagnosis	25OHD is a good marker of vitamin D status but serum calcium is a poor marker of calcium status. Consistent and early biochemical markers for diagnosis of rickets and osteomalacia are elevations in serum ALP and PTH. The diagnosis of rickets requires radiological confirmation.
High prevalence in risk groups	NR/osteomalacia is less common in the white population but a common disease in ethnic risk groups with dark skin or cultural full body clothing, including refugees. These groups require lifelong supplementation and/or food fortification programmes.
Measuring 25OHD and indication for supplementation	Measuring 25OHD is not required in asymptomatic individuals. Instead, lifelong supplementation should be recommended based on ethnicity, culture and other risk factors for calcium deprivation.
Prevention and supplementation	Universal supplementation of pregnant women and infants with vitamin D is an essential public health strategy, as the foetus and infant have a human right to be protected against harm. The recommendation is now to supplement all infants regardless of skin colour or feeding status with 400 IU/day in the first year of life and longer in those with persistent risk factors (i.e. dark skin).
Micronutrient deficiencies rarely occur in isolation	In high-risk groups and malnourished individuals, vitamin D deficiency often occurs combined with other micronutrient deficiencies, in particular iron and folate deficiency.

#### Identifying Successful and Sustainable Implementation Strategies

Although most developed countries have vitamin D supplementation policies in place, some countries lack successful implementation strategies. Recent studies from Canada and New Zealand reported that none of the individuals with NR had received vitamin D supplements for rickets prevention despite the presence of national recommendations [69, 70]. Similarly, 85% of British parents are unaware of the need for infant vitamin D supplementation for their baby despite existing policy [71]. There is overwhelming evidence that infant vitamin D supplementation improves 25OHD concentrations [72] and prevents NR [73]. We recently studied policy features and implementation strategies that influence good adherence to supplements (≥80% of infants at 1 year) [61]. Although 97% of European countries studied (*n* = 29) had a national supplementation policy in place, policy features varied. Efficient implementation strategies such as monitoring adherence at child health surveillance visits (*p* = 0.001), universal supplementation (*p* = 0.007), providing information to families at discharge from neonatal units (*p* = 0.02) and financial family support (*p* = 0.005) were lacking in countries with low adherence (<50% of infants). Moreover, countries with high adherence adopted more policy factors and implementation strategies compared to moderate and low adherence countries (*p* < 0.001) [61].

Policy makers should clarify the responsibilities of healthcare professionals delivering the policy [4•] such as providing information on prevention, prescribing supplements and checking adherence at child health surveillance or immunisation visits. Interestingly, free supply of vitamins alone does not automatically enhance adherence and participation [74, 75].

#### Health Promotion

In 2008, Turkey reduced the incidence of NR by visiting primary health stations throughout the country and supplying free vitamins [73]. However, a recent survey demonstrated persistent poor knowledge among healthcare professionals of existing prevention programmes in Turkey [76]. Similar health promotion campaigns have proven successful [77, 78]; however, the results are not sustained [3]. Therefore, more sustainable delivery of information to both healthcare professionals and target groups is essential. The global consensus group recommends integration of vitamin D supplementation into antenatal and infant child care passport programmes [13••, 14]. Such programmes should dictate monitoring of adherence and prescription of supplements at all prenatal and child health surveillance visits. Bolus vitamin D doses alongside vaccinations has been done successfully [79].

#### Food Fortification with Vitamin D or Calcium

Health benefits of food fortification with micronutrients, including vitamin D, are well established [80]. Currently, most European countries follow voluntary fortification [81]; however, evidence from Canada and the USA suggests that mandatory fortification improves vitamin D uptake at the population level [82]. Fortification should not be restricted to dairy products due to its limited consumption in certain risk groups [83]; fortification of a variety of food sources is more beneficial [81]. Countries with established mandatory fortification are now adopting novel approaches such as ‘bio-addition’ which involves fortification of staple food through addition of vitamin D-rich food to animal feed during production or manipulation of food post-harvest or pre-processing [82]. In order to address malnutrition and the multiple micronutrient deficiencies across the globe, it is crucial that governments and international agencies look beyond single micronutrient deficiencies and initiate mandatory fortification [84, 85]. For regions with traditionally low dietary calcium, e.g. Africa [86] and India [87], the global consensus group recommended use of calcium supplement from indigenous sources [13••, 14].

## Areas Warranting Further Research

### The True Prevalence of NR and Osteomalacia

The true prevalence of NR and osteomalacia across the globe remains unknown [88]. Studies are urgently needed to establish their prevalence at a population level, in particular in areas where such data would matter most for public health, as in Africa, the Indian subcontinent and the Middle East. Most population screening studies to date have used clinical signs, biochemical markers (raised ALP and PTH, low calcium, low 25OHD) and radiological signs to define rickets, either in isolation or in combination [89]. Further studies are warranted to identify the most appropriate biochemical marker for screening of NR. Ultimately, the *histological presence of osteomalacia* defines the presence of pathology, and research should focus on how histology relates to levels of calcium deprivation, elevations of ALP and PTH, radiological signs of rickets and fracture risk (Fig. 1). Until a reliable screening tool is available, NR should be considered a reportable disease by health authorities.

### Efficacy and Safety of Targeted Intermittent Oral Supplementation and Fortification Programmes

A lot more work needs to be done on trials designed to demonstrate safety and efficacy of community-based, targeted supplementation programmes, including adherence and health economics. Similarly, food fortification programmes, ideally incorporating multiple nutrients such as folic acid, require large scale trials.

### Vitamin D Deficiency and Non-accidental Injuries

The role of vitamin D deficiency (with absent radiological signs of rickets) in bone fragility is much debated in the context of non-accidental injuries [90, 91]. The state of pre-rickets/osteomalacia **(**Fig. 1 **)** is only identified on bone histology. It has been demonstrated that the pickup rate of biopsy proven rickets by radiologists is poor [8•, 9, 92]. NR and osteomalacia, as hypophosphataemic complications, occur late in the time course of calcium deprivation, and thus radiological signs occur very late. It is only reasonable to assume that histological and histomorphometric changes precede x-ray changes [4•]. No studies have tested bone fragility in the setting of *low 25OHD levels (with or without ALP/PTH elevation) with normal radiology of the growth plate*. Therefore, despite association studies [93–95], there is currently insufficient evidence to conclude that the child is susceptible to fractures in this setting. Only a well-designed bone biopsy study would help answer this question.

### Undiagnosed NR and Mortality

Studies describe incidental cardiomyopathy and histological evidence of NR in Sudden Unexpected Death in infancy and childhood [8•, 9]. Prolonged severe vitamin D deficiency leads to hypocalcaemia which can cause sudden death in infants [8•]. The prevalence of insufficient 25OHD levels in undiagnosed cases of sudden infant death at post-mortem is high [9]. Further, large scale, prospective studies are required to confirm the true prevalence of NR and osteomalacia, which includes establishing the feasibility of measuring 25OHD, and markers of other micronutrient deficiencies, post-mortem.

### Genetic Polymorphisms and Best Marker of Vitamin D Status

Polymorphisms in genes encoding key enzymes (i.e. CYP3A4, CYP24 and CYP27B1) and proteins that affect the circulating concentrations of vitamin D metabolites have been described [96]. Polymorphisms in the genes encoding vitamin D binding protein help to explain the increased susceptibility of African American infants and toddlers to vitamin D deficiency [97]. However, only less than 5% of the variations in serum 25OHD concentrations are explained by genetic variants [96] with major determinants still being lifestyle factors such as exposure to sunlight and dietary habits. Vitamin D status is universally determined by 25OHD measurements; however, the search for the ideal biochemical marker continues [98].

## Conclusions

The global rise in a preventable disease like NR in the twenty-first century is unacceptable. NR is a common disease in high-risk groups worldwide, even in high income countries, highlighted by refugee crises and changing population demographics. However, fair-skinned people are not exempt from risk. The global consensus recommendations [13••, 14] provide guidance both for clinicians and policy makers on the prevention of rickets, and infants, pregnant women and dark-skinned individuals should be at the core of any policies. In addition to implementing successful supplementation programmes for these risk groups, mandatory food fortification should be considered by government bodies and international agencies as a means to improve nutritional vitamin D and/or calcium status at the population level. Areas that warrant further public health research include the ideal marker for screening of NR and osteomalacia as well as for vitamin D status, intermittent oral supplementation administered by healthcare providers and the relationship between early signs of a mineralization defect (i.e. bone biochemical or histological findings) on bone fragility and on infant and child mortality.
